# Introduction of NGS in Environmental Surveillance for Healthcare-Associated Infection Control

**DOI:** 10.3390/microorganisms7120708

**Published:** 2019-12-16

**Authors:** Manola Comar, Maria D’Accolti, Carolina Cason, Irene Soffritti, Giuseppina Campisciano, Luca Lanzoni, Matteo Bisi, Antonella Volta, Sante Mazzacane, Elisabetta Caselli

**Affiliations:** 1 Institute for Maternal and Child Health-IRCCS “Burlo Garofolo”, 34137 Trieste, Italy; mcomar@units.it (M.C.); casoncarolina@gmail.com (C.C.); giusi.campisciano@burlo.trieste.it (G.C.); 2 Department of Medical Sciences, University of Trieste, 34137 Trieste, Italy; 3 Section of Microbiology, Department of Chemical and Pharmaceutical Sciences, University of Ferrara, 44121 Ferrara, Italy; maria.daccolti@unife.it (M.D.); irene.soffritti@unife.it (I.S.); 4 CIAS (Centro ricerche Inquinamento ambienti Alta Sterilità) Research Center, University of Ferrara, 44122 Ferrara, Italy; luca.lanzoni@unife.it (L.L.); matteo.bisi@unife.it (M.B.); antonella.volta@unife.it (A.V.); sante.mazzacane@unife.it (S.M.)

**Keywords:** healthcare-associated infections, antimicrobial resistance, contamination, molecular methods, next generation sequencing

## Abstract

The hospital environment significantly contributes to the onset of healthcare associated infections (HAIs), representing the most frequent and severe complications related to health care. The monitoring of hospital surfaces is generally addressed by microbial cultural isolation, with some performance limitations. Hence there is need to implement environmental surveillance systems using more effective methods. This study aimed to evaluate next-generation sequencing (NGS) technologies for hospital environment microbiome characterization, in comparison with conventional and molecular methods, in an Italian pediatric hospital. Environmental samples included critical surfaces of randomized rooms, surgical rooms, intensive care units and delivery rooms. The resistome of the contaminating population was also evaluated. NGS, compared to other methods, detected with higher sensitivity the environmental bacteria, and was the only method able to detect even unsearched bacteria. By contrast, however, it did not detect mycetes, nor it could distinguish viable from dead bacteria. Microbiological and PCR methods could identify and quantify mycetes, in addition to bacteria, and PCR could define the population resistome. These data suggest that NGS could be an effective method for hospital environment monitoring, especially if flanked by PCR for species identification and resistome characterization, providing a potential tool for the control of HAI transmission.

## 1. Introduction

Healthcare-associated infections (HAIs) represent one of the most frequent and severe complications of hospitalization worldwide, as 5%–15% of all hospitalized inpatients acquire them during hospitalization [1]. In Europe, more than 4 million of patients develop HAIs each year [2,3], and in Italy the incidence of infectious complications generally varies from 5%–10%, with a mortality rate of 20%–30% [4]. HAIs are known to be responsible for significant excess morbidity and mortality, lengthening the duration of stays, as well as imposing additional costs on health systems [5]. They are frequent in several ward types, including pediatric wards, as children are particularly susceptible to infections due to several factors, including an immature immune system, the presence of acquired or congenital immunodeficiencies, effects caused by anti-cancer therapy, etc. [6].

In addition, antimicrobial resistance (AMR) is currently spreading worldwide, representing one of the major threats for human health, and is particularly dangerous in healthcare settings, where antimicrobials are heavily used, exerting an important selective pressure on microbes. Not surprisingly, AMR is strongly associated with HAI severity, as many HAIs are caused by multidrug resistant (MDR) or even pan-drug resistant (PDR) microbes [7,8].

Persistent contamination of hospital surfaces by potential pathogens significantly contributes to HAI onset, being an environmental reservoir for several microbes, including methicillin-resistant *Staphylococcus aureus* (MRSA), vancomycin-resistant enterococci (VRE), *Clostridium difficile, Pseudomonas aeruginosa*, *Acinetobacter* spp., as well as viruses [9,10,11,12]. It is generally accepted that hospital contamination is associated with HAI transmission [13,14], as the risk of acquiring an infection caused by a specific microbe increases for a person admitted in a hospital room that was previously occupied by a patient colonized or infected by the same infectious agent [15]. This is due to the ability of resilient microorganisms to survive for long period on surfaces [16], from where they can be easily transmitted to other patients [14], or shed in other rooms to unaffected patients by horizontal transmission from asymptomatic carriers, such as healthcare workers or visitors [17].

Therefore, it is clear that environmental cleaning in hospitals has a crucial role in reducing microbial contamination of surfaces and the subsequent risk for HAIs. Scientific evidence reports that an adequate and correct cleanliness, accompanied by specific campaigns to raise awareness for importance of hand hygiene, is associated with a reduction of surface contamination and a concomitant decrease of pathogen transmission to patients [12,18].

In this regard, we recently showed that abating microbial contamination and its AMR in the hospital environment, using a cleaning procedure, was associated with a significant reduction of HAI incidence [19] suggesting that it could be used as a potential strategy for infection control and prevention.

Defining the hygiene of the healthcare setting environment is not simple. A solely visual assessment provides only a rough indication of “cleanliness”, and cannot predict the real infection risk for patients [10,20]. From this perspective, specific methods able to assess the microbial presence in the hospital environment represent an important point for the control of pathogenic contamination and infection prevention. Until now, monitoring of hospital environmental surfaces took place by microbiological, culture-based methods [21,22], which however show important limitations mostly linked to the need for culture isolation and culture time [22]. Indeed, although being able to detect viable bacteria or fungi, these methods can detect only known microbes, by using specific selective media, need long incubation times and specific conditions (temperature and atmosphere) to allow microorganisms to grow, rendering them very time-consuming and complex when used for the analysis of a whole microbial population [22].

By contrast, an efficient environmental monitoring system should be precise and rapid, able to provide a detailed characterization of the bioburden as soon as possible. In this regard, the assessment of the effectiveness of cleaning procedures performed by measurement of adenosine triphosphate (ATP) by bioluminescence, is a rapid method, but has low accuracy since it can also detect organic materials, providing potential false positive results [23]. Furthermore, it cannot distinguish between pathogenic and non-pathogenic microorganisms. Hence the urgency for implementing environmental surveillance systems to characterize environmental bioburden.

Molecular methods based on DNA technologies might overcome such limitations, providing accurate information in real time and helping to characterize in detail the whole microbial population colonizing hospital surfaces. To address this aim, specific PCRs and real-time PCRs have been set up and developed [19,22,24,25]. However, the advent of modern technologies based on DNA sequencing has considerably improved microbiome investigation allowing definition of complex populations in deep detail [26], and could also be employed for environmental surveillance [22].

Lastly, several studies have been performed to characterize the microbial contamination in the hospital wards hosting adult patients, but considerably less is known about the contamination level and type in paediatric healthcare settings.

Based on these considerations, the present study aimed to characterize the contamination in an Italian paediatric hospital, by using next generation sequencing (NGS) technologies in comparison with conventional microbiological and molecular PCR methods, trying to define surface microbiomes and to characterize advantages and disadvantages of the compared technologies.

## 2. Materials and Methods

### 2.1. Environmental Sampling

The study was conducted after obtaining approval from the Institutional Scientific Board of the Institute for Maternal and Child Health “IRCCS Burlo Garofolo” (Trieste, Italy) in the following wards: Pediatric Clinic (PC), Pediatric Surgery (PS), Pediatric Oncology (PO), Neonatal Intensive and Sub-Intensive Care Unit (NICU and NICUs), children’s Intensive Care Unit (ICU), Surgical Rooms (SR: Orthopedics, Gynecology and Oculistics) and Delivery Room (DR).

Two sampling campaigns were performed for each enrolled ward, in which up to three rooms/wards were monitored. Environmental samples were collected seven hours after cleaning, a time chosen based on our previous studies on hospital contamination, showing that this time point is representative of the contamination level in a 24-h period [19,25,27]. Different hard surfaces were sampled based on the characteristics of the individual wards: floor for all ward types; bed footboard, operating bed or incubator for inpatients wards, ICU, SR and NICU, respectively; sink or operating table for inpatients rooms or SR/DR, respectively. The same points were simultaneously sampled following two different methodologies according to subsequent microbiological or molecular analyses.

### 2.2. Microbiological Analyses

For microbiological analyses, sampling was performed in duplicate by using Replicate Organism Detection and Counting (RODAC) contact plates containing the following specific culture media: total bacteria (TSA medium, Merck Millipore, Milan, Italy), *Staphylococcus* spp. (Baird Parker medium, Merck Millipore, Milan, Italy), Enterobacteriaceae spp. (MacConkey medium, Merck Millipore, Milan, Italy), *Acinetobacter* spp. (Herella medium, Lickson, Milan, Italy), *Pseudomonas* spp. (Cetrimide medium, Incofar, Modena, Italy), *Clostridium difficile* (*Clostridium difficile* selective medium, Lickson, Milan, Italy), *Enterococcus* spp. (BEA medium, Incofar, Modena, Italy) and mycetes (Sabouraud medium, Merck Millipore, Milan, Italy). Plates for general or selective growth of bacteria were incubated at 30 °C for 24–48 h (respectively for general and selective media), whereas plates for the specific growth of mycetes were incubated at 25 °C for 72 h. Colony forming units (CFU) were then enumerated. Plates containing ≥ 200 CFUs were considered to have 200 CFUs, following the guideline INAIL-2017 [28]. A total of 216 samples were collected from surfaces.

### 2.3. Molecular Analyses

For molecular analyses, 108 total environmental samples representing total microbial population were collected by sterile rayon swabs rubbed on a 10 × 10 cm area as previously described [25]. Swabs were pre-moistened in sterile Tryptic Soy Broth (TSB) or saline, depending on the subsequent analysis type, and rubbed swabs were put in 5 mL TSB or 0.4 mL saline respectively, immediately refrigerated and transported to the laboratory.

Samples collected in TSB were incubated at 37 °C for 24 h for a controlled bacterial amplification; afterwards microbial cells were pelletized (14,000 × *g* for 5 min) and stored until use at −20 °C as already described [19]. Microbial DNA was extracted from microbial pellets by the QIAmp UCP Pathogen Mini Kit (Qiagen, Hilden, Germany), and analyzed by the Microbial DNA qPCR Array for Antibiotic Resistance Genes (Antibiotic Resistance Genes, catalog no. BAID-1901ZRA; Qiagen), allowing the detection and quantitation of 84 genes coding for antimicrobial resistance, as previously described [25,27]. An amount of 1 µg of extracted DNA per plate (10 ng/well/reaction) was used for R gene microarray analysis.

Samples collected in saline were immediately frozen at −80 °C, without amplification, and used to quantify microbial communities in sampling real time. After being thawed and vortexed, total DNA was extracted from 300 µL of each sample by the Exgene Cell SV Kit (Gene All, Tema Ricerca, Bologna, Italy), in a final elution volume of 100 µL. DNA samples were then analyzed by a customized qPCR microarray (BAID-00047RA Qiagen, Hilden, Germany), assessing simultaneously the presence of the following microbes: *S. aureus, S. epidermidis, E. faecalis, E. faecium, E. coli, K. pneumonia/Enterobacter, A. baumannii, P. mirabilis, P. aeruginosa, C. perfringens, C. difficile, A, fumigatus and C. albicans*, as previously reported [29]. An amount of 120 ng per sample (corresponding to 7 ng of template DNA per well/reaction) was used for the custom-array analysis.

### 2.4. NGS Analyses

For NGS analyses, environmental samples were collected as described for qPCR molecular assays, then total DNA was extracted in a final elution volume of 50 µL by the automatic extractor Maxwell CSC DNA Blood Kit (Promega, Madison, WI, USA), according to manufacturer’s instruction. DNA samples were stored at −80 °C prior to further processing.

The characterization of the bacterial composition of the samples was performed by sequencing the region V3 of the 16S rRNA gene. First, a qPCR targeting the V1–V3 region 16S rRNA gene (500 bp), was performed using EvaGreen^®^ dye (Fisher Molecular Biology, Waltham, MA, USA), by employing the U534R primer (U534R 5′-ATTACCGCGGCTGCTGG-3′) and the degenerated primer 27FYM (5′-AGR GTT YGA TYM TGG CTC AG - 3′). A nested PCR was subsequently carried out with the primers B338F_P1-adaptor (B338F 5′-ACTCCTACGGGAGGCAGC-3′) and U534R_A_barcode, targeting the V3 region (200 bp) of the 16S rRNA gene, with a different barcode for each sample linked to the reverse primer [30]. The analysis was simultaneously performed on negative controls, including no DNA template. The PCR reactions were performed using the Kapa 2G HiFi Hotstart ready mix 2X (Kapa Biosystems, Wilmington, MA, USA) and BSA 400 ng/μL, under the following temperature cycling conditions: 95 °C for 5 min, 95 °C for 30 sec, 59 °C (V1–V3 PCR)/57 °C (V3 PCR) for 30 sec, and 72 °C for 45 sec with a maximum of 27 cycles for the V1–V3 PCR and a maximum of 13 cycles for the V3 PCR. Final elongation step was of 10 min at 72°C.

After assessing the correct size of the amplicon (260 bp) on a 2% acrylamide gel, the DNA quantification was performed by a Qubit^®^ 2.0 Fluorimeter (Invitrogen, Carlsbad, CA, USA) and an equal amount of each sample was mixed to generate a pooled library at a final 100 pM concentration, according to manufacturer’s instructions. The Ion OneTouch™ 2 System (Life Technologies, Gran Island, New York, USA) was used to prepare the template with the Ion PGM Hi-Q View OT2 200 kit (Life Technologies, New York, USA). Sequencing was performed with the Ion PGM™ System technology by using the Ion PGM Hi-Q View sequencing kit (Life Technologies, New York, USA). Quantitative Insights Into Microbial Ecology (QIIME 1.9.1.) software, allowing analysis of high-throughput community sequencing data [31] (available at: https://www.nature.com/articles/nmeth.f.303; accessed: 1st October 2019) was used to process the sequence data. High quality sequences (Q > 25) were demultiplexed and filtered by quality using split_libraries_fastq.py with default parameters, except for the length (150 bp). Sequences with homopolymer length >8 or ambiguous bases were removed. Operational Taxonomic Units (OTUs) were clustered with a similarity threshold of 97% and aligned against the reference taxonomy database SILVA V.132 [32].

### 2.5. Statistical Analyses

Statistical analyses were performed using parametric Student’s *t*-test and assuming as statistically significant a P value at least <0.05. Bonferroni correction for multiple comparisons was applied for analysis of microarray data (a Pc value <0.05 was considered significant). NGS data were statistically analyzed by QIIME 1.9.1. Beta diversity, assessed by weighted and unweighted UniFrac distance matrices [33], and presented with principal coordinates analysis (PCoA). Analyses of Similarities (ANOSIM) was performed to compare the composition of microbial community among the tested ward surfaces.

## 3. Results

### 3.1. Environmental Analyses by Conventional Microbiology

In order to characterize the environmental microbial contamination in a pediatric Italian hospital, different wards were sampled and analyzed in parallel by conventional microbiological as well by molecular methods. Areas included: Pediatric Clinic (PC), Pediatric Surgery (PS), Pediatric Oncology (PO), Neonatal Intensive and sub-Intensive Care Unit (NICU and sNICU), children’s Intensive Care Unit (ICU), Surgical Rooms (SR: Orthopedics, Gynecology and Oculistics) and Delivery Room (DR) were included in the analyses. Each ward was sampled in two consecutive sampling campaigns, and up to three rooms per ward were tested. The following critical surfaces were sampled, as they are particularly representative of hospital contamination based on previous results [19,27]: floor, bed footboard and sink for inpatients wards and ICU; floor, operating bed and operating table for SR and DR; floor, incubator and sink for NICU. Each sample was collected in duplicate by applying Replicate Organism Detection and Counting (RODAC) contact plates directly to the sampled point. RODAC contained different general and selective media allowing detection and quantification of the following HAI-related microorganisms: total bacteria (TSA medium), *Staphylococcus* spp. (Baird Parker selective medium), *Enterobacteriaceae* spp. (MacConkey selective medium), *Acinetobacter* spp. (Herella selective medium), Mycetes (Sabouraud selective medium), *Pseudomonas* spp. (Cetrimide selective medium), *Clostridium difficile* (*Clostridium difficile* selective medium), and *Enterococcus* spp. (BEA selective medium). Plates were incubated as described in the Materials and Methods section based on the type of microorganism, and colony forming units (CFU) were enumerated. A total of 216 microbiological samples (108 surfaces in duplicate) were collected and analyzed.

**Figure 1:**
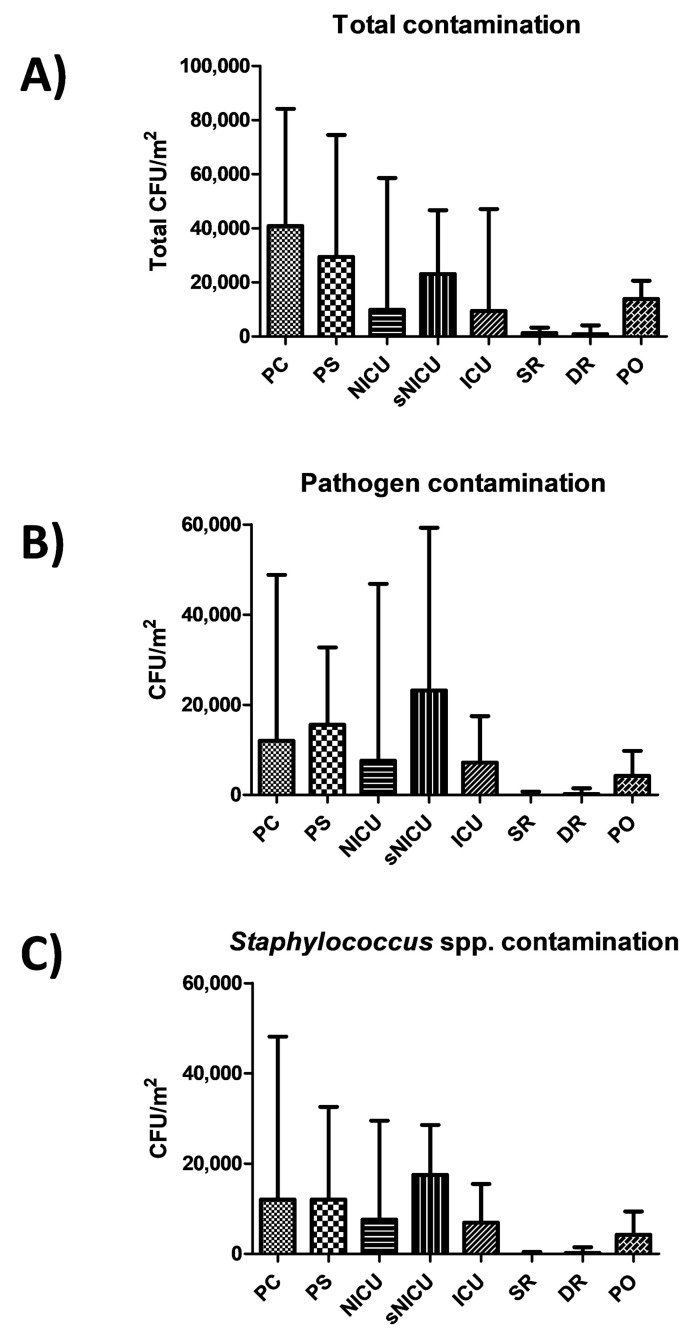
Microbial contamination in wards analyzed by conventional culture-based methods. (**A**) Total contamination, corresponding to CFUs obtained on TSA medium; (**B**) pathogen contamination, corresponding to the sum of the searched pathogens (CFUs obtained on selective media); (**C**) staphylococcal contamination, corresponding to CFUs obtained on selective BP medium. All results are expressed as median value of CFU/m^2^ with interquartile range. PC, Pediatric Clinic; PS, Pediatric Surgery; NICU, Neonatal Intensive; sNICU, sub-Intensive Care Unit; ICU, children’s Intensive Care Unit; SR, Surgical Rooms; DR, Delivery Room; PO, Pediatric Oncology.

Results showed a diverse level of contamination in the different wards, as expected (Figure 1A). Most of contamination was ascribable to potential pathogens (Figure 1B), largely represented by *Staphylococcus* spp. (Figure 1C). In particular, PC was the ward with the highest level of contamination, having a median total CFU count corresponding to 40,842 CFU per square meter (95% CI 30,412–54,664). A considerable proportion of contaminants were represented by the searched pathogens (including *Staphylococcus* spp., *Enterobacteriaceae*, *Klebsiella* spp., *Acinetobacter* spp., *Pseudomonas* spp., *Clostridium difficile*, *Enterococcus* spp., *Candida* spp., *Aspergillus* spp.), whose sum resulted in a median value of 12,000 CFU/m^2^ (95% CI 2526–40,545), representing 69.5% of the total mean microbial contamination. Pathogen contamination was highly referable to *Staphylococcus* spp. (median CFU 12,985/m^2^, 95% CI 2526–39,662). PS had a similar value of contamination, with a total median value of 29,474 CFU/m^2^ (95% CI 25,762–47,619), and with a median value of pathogens corresponding to 15,579 CFU/m^2^ (95% CI 2631–31,580), highly represented by *Staphylococcus* spp. (median 12,000 CFU/m^2^, 95% CI 2105–30,188).

Rooms of sNICU ward had levels of contamination similar to PS, with a median value of total contaminants corresponding to 23,158 CFU/m^2^ (95% CI 19,387–38,485), the sum of pathogens corresponding to a median value of 23,158 CFU/m^2^ (95% CI 1579–42,515), and the Staphylococcal median contamination at 17,474 CFU/m^2^ (95% CI 1263–27,673). The PO ward showed a median total count of 13,894 CFU/m^2^ (95% CI 11,948–26,392), with a median pathogen level corresponding to 4210 CFU/m^2^ (95% CI 28,56–13,354), mostly referable to *Staphylococcus* genus (median value 4210 CFU/m^2^; 95% CI 526–13,224). Compared to the other wards, ICU and NICU showed a considerably lower degree of contamination, having respectively a median total value of 9474 CFU/m^2^ (95% CI 5234–45,082) and 9895 CFU/m^2^ (95% CI 6.346–48,882). The median value of the sum of the ten searched pathogens corresponded to 7158 CFU/m^2^ in ICU (95%CI 1053–17,723), and to 7579 CFU/m^2^ in NICU (95% CI 105–57,343). In both wards, staphylococci represented the majority of pathogenic contaminants, corresponding respectively to 6947 CFU/m^2^ in ICU (median value; 95% CI 1053–16,871), and 7579 CFU/m^2^ (median value; 95% CI 105–26,435) in the NICU ward. As expected, SR and DR showed a very low level of total microbial contamination, corresponding respectively to a median value of 1263 CFU/m^2^ (95% CI 1459–5348) and 842 CFU/m^2^ (95% CI 0–8793). The sum of pathogens corresponded respectively to a median value of 0 CFU/m^2^ in SR (95% CI 0–1842), and 210 CFU/m^2^ in DR (95% CI 0–2889), with a median amount of staphylococci corresponding to 0 CFU/m^2^ in SR (median value; 95% CI 0–1821), and 210 CFU/m^2^ in DR (median value; 95% CI 0–2889).

No statistically significant differences emerged among PC, PS and sNICU, whereas PO, NICU and ICU were significantly less contaminated (*p* < 0.01). SR and DR, as expected, were significantly different both compared to the PC–PS–sNICU group (*p* < 0.0001) and to the PO–NICU–ICU group (*p* < 0.001).

**Figure 2:**
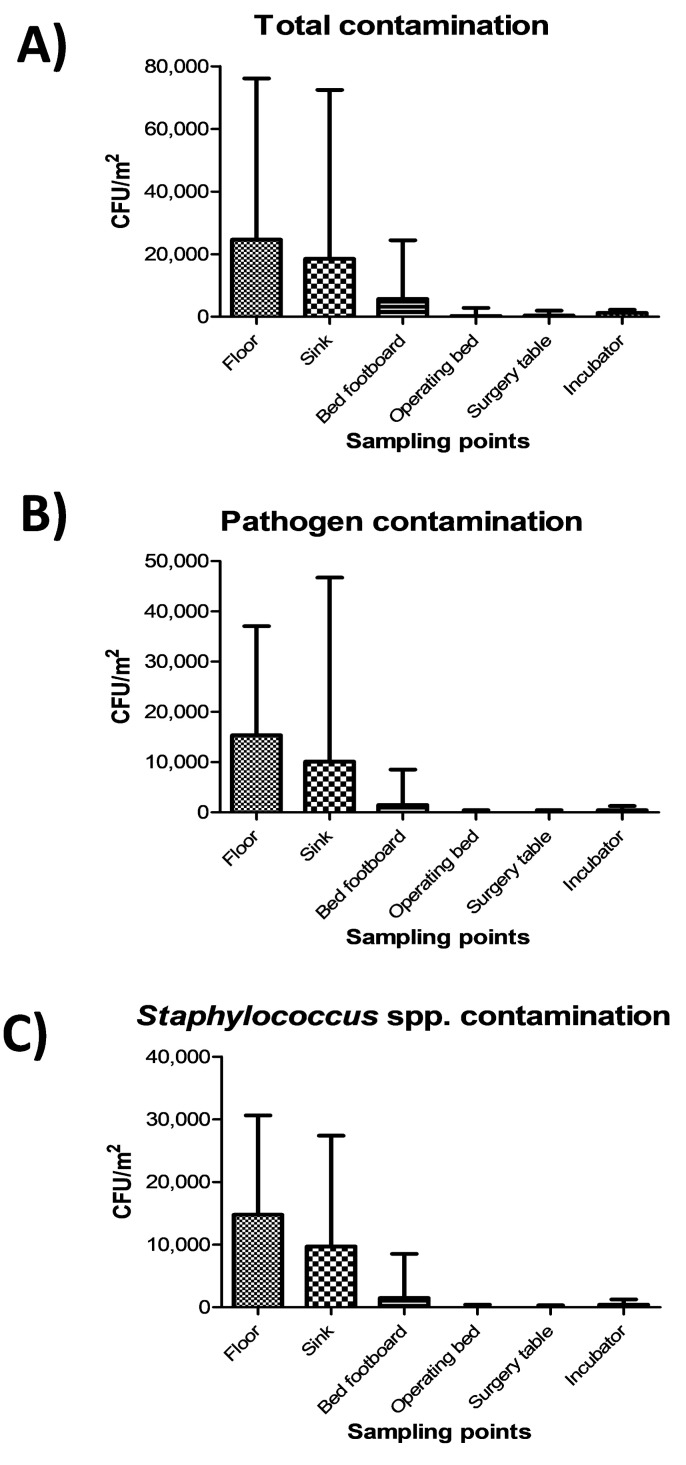
Microbial contamination on analyzed surfaces by conventional culture-based methods: (**A**) Total contamination, corresponding to CFUs obtained on TSA medium; (**B**) Pathogen contamination, corresponding to the sum of CFUs of the searched pathogens on selective media; (**C**) Staphylococcal contamination, corresponding to CFUs obtained on selective BP medium. All the results are expressed as the median value of CFU/m^2^ with interquartile range.

The highest level of contamination was found on floor, and similarly high values were also detected in sinks (Figure 2A) (*p* = ns). Bed footboards resulted significantly less contaminated (*p* < 0.01), and minimal levels of microbial contamination were detected, as expected, on the other tested surfaces (operating beds, surgery tables and incubators) (*p* < 0.0001). Most microbial contamination consisted in potential pathogens, as the sum of the ten searched pathogens represented up to 90% of the total contamination (Figure 2B), mostly represented by *Staphylococcus* spp. (Figure 2C).

**Figure 3:**
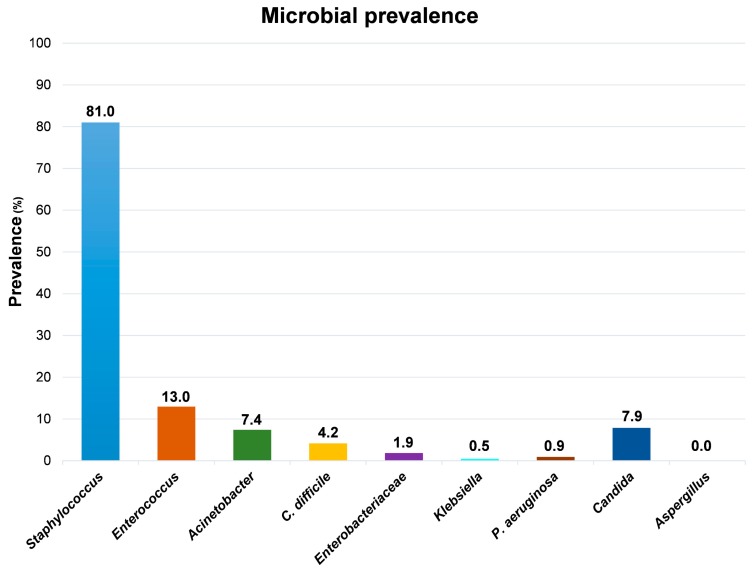
Main genera/species/families detected on tested surfaces by conventional culture-based methods. Results represent the percentage of detection of the indicated genera/species/families on the total collected samples (216 samples).

Although the high level of contamination ascribable to the *Staphylococcus* genus, which was detected in 175/216 samples (81%), the species *S. aureus* was identified uniquely in one out of all the collected samples, suggesting that the vast majority of staphylococci contaminating the tested surfaces were coagulase-negative strains. Compared to *Staphylococcus* spp., the other searched microbial genera appeared much less represented (Figure 3).

*Enterococcus* spp., representing the most prevalent contaminant genus after *Staphylococcus,* were detected in 28 samples, exclusively from floors and sinks, mostly from PC rooms and partly from sNICU and PO wards. *Acinetobacter* spp. was isolated from 16 sampled surfaces, prevalently from floors and sinks from ICU, sNICU and PS wards. *C. difficile* was evidenced in 9 samples from floors and sinks of PC and sNICU, whereas Enterobacteriaceae were generally scarcely detected, being isolated in only 4 samples (2 sinks and 2 floors from PC and PS wards), concomitantly with *Klebsiella* spp. in one of the sinks samples. Similarly, *P. aeruginosa* was identified uniquely in two sink samples from PC rooms. With regard to mycetes, *Candida* spp. were evidenced in 17 samples from sinks of PC and sNICU wards, whereas *Aspergillus* spp. were never detected.

### 3.2. Environmental Analyses by Molecular Assay (qPCR Microarray)

**Figure 4:**
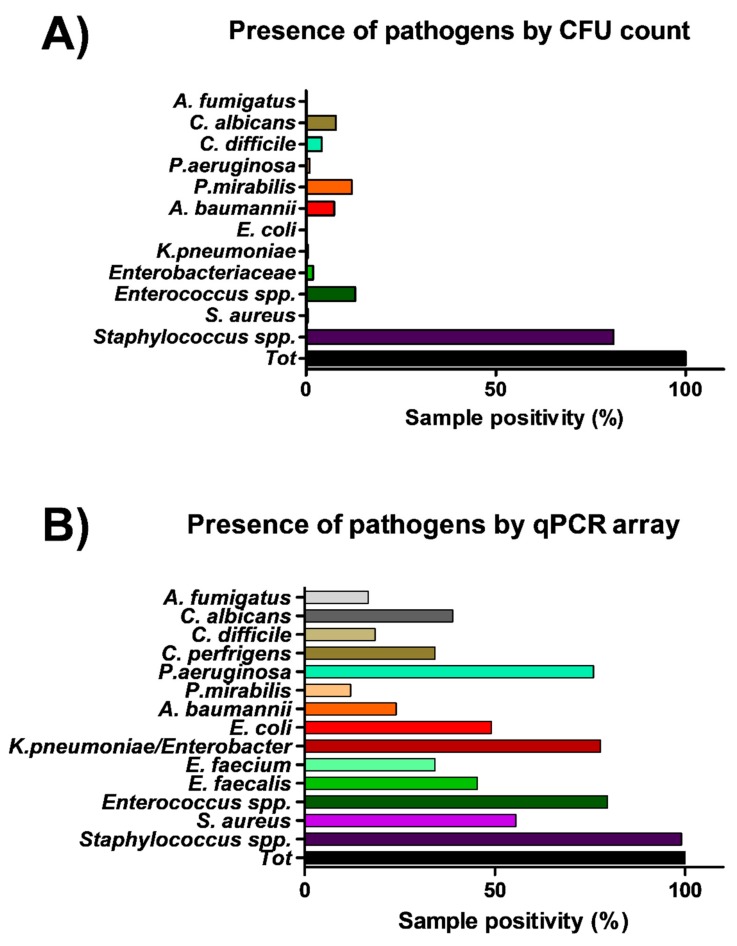
Comparison of culture-based and molecular methods in the efficiency of detection of microbial contamination in all analyzed samples. (**A**) Culture-based methods (216 total samples). (**B**) Molecular qPCR assay (108 total samples). Results represent the percentage of detection of the indicated families/genera/species on the total collected samples.

The same points sampled for microbiological analysis were in parallel collected by moistened swabs rubbed on a 10 × 10 cm surface for molecular analysis. A total of 108 total swab samples were collected, from which total DNA was extracted and analyzed by a quantitative real-time PCR (qPCR) customized array, assessing simultaneously the presence of the following bacterial and mycotic species: *S. aureus, S. epidermidis, E. faecalis, E. faecium, E. coli, K. pneumonia/Enterobacter, A. baumannii, P. mirabilis, P. aeruginosa, C. perfringens, C. difficile, A, fumigatus and C. albicans*. Total bacterial and mycetes DNA was also assessed in parallel, respectively by a pan-bacterial and pan-mycetes qPCRs included in the array as a control. The results allowed the identification of the searched pathogens in more surface samples compared to conventional methods (Figure 4). In fact, genus *Staphylococcus* was identified in 107 out of 108 samples (99%), including 60 samples positive for the presence of *S. aureus* (55%). *Enterococcus* genus was detected in 86 samples, of which 49 harbored *E. faecalis* and 37 *E. faecium*, respectively. Bacteria belonging to the *Enterobacteriaceae* family were evidenced in many samples, compared to what was detected by microbiological analysis, as *K. pneumonia/Enterobacter* was evidenced in 84/108 collected samples, often in association with *E. coli* (in 53 samples), *A. baumannii* (in 26 samples), *P. mirabilis* (in 13 samples), *P. aeruginosa* (in 82 samples), *C, perfringens* and *difficile* (in 37 and 20 samples, respectively). Also searched-for mycetes were quite represented in surface microbiomes, as *C. albicans* and *A. fumigatus* species were detected in 42 and 18/108 environmental samples, respectively.

**Figure 5:**
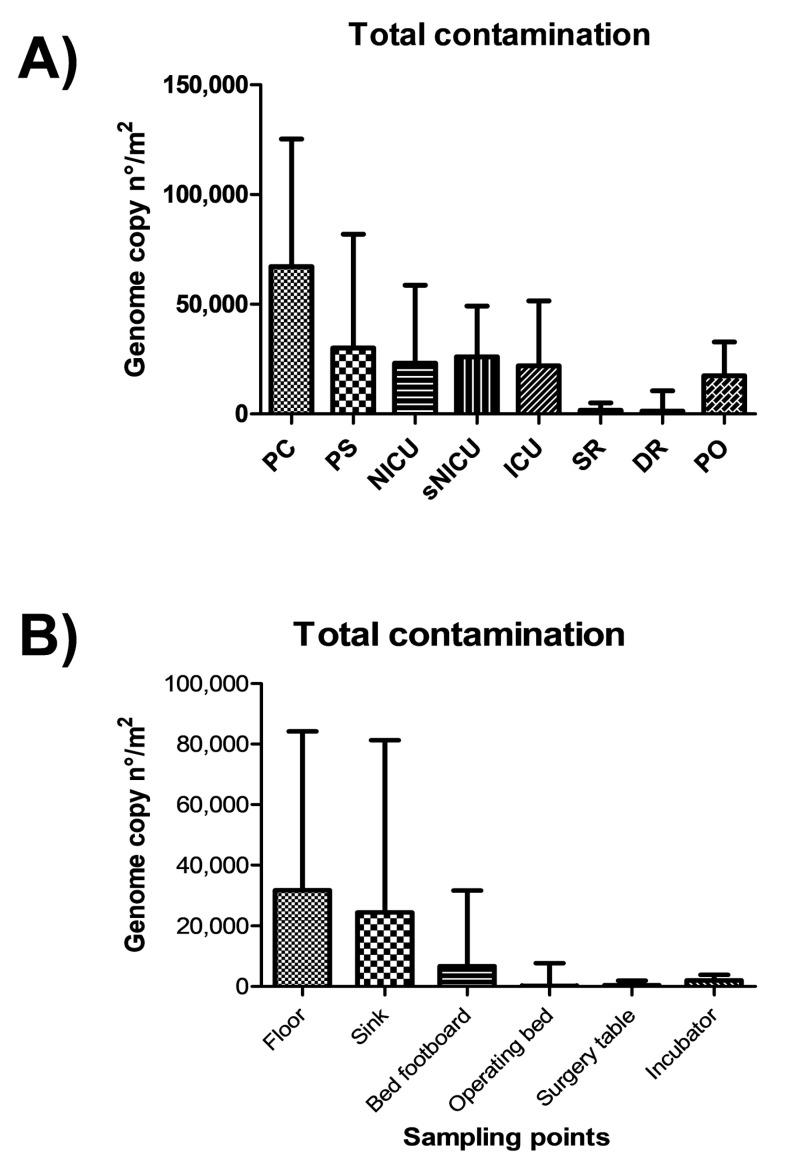
Quantitation of microbial contamination by qPCR molecular assays. (**A**) Microbial contamination in the analyzed wards, expressed as median value of genome copy number/m^2^, with interquartile range. (**B**) Microbial contamination on the analyzed surfaces, expressed as median value of genome copy number/m^2^, with interquartile range. PC, Pediatric Clinic; PS, Pediatric Surgery; NICU, Neonatal Intensive; sNICU, sub-Intensive Care Unit; ICU, children’s Intensive Care Unit; SR, Surgical Rooms; DR, Delivery Room; PO, Pediatric Oncology.

The highest levels of contamination were observed in the PC and PS wards, as judged by summing up the genome copy amounts measured by qPCR, whereas SR and DR were less contaminated, confirming what was observed by conventional microbiological analyses (Figure 5). Statistically significant differences were observed between PC–PS wards and the group of wards NICU–sNICU–ICU–PO (*p* < 0.05). SR and DR were significantly different from both the first (*p* > 0.0001) and the second group of wards (*p* < 0.001).

On average, the measurement of genome copy number yielded higher values of contamination per square meter, compared to CFU counts. This result might be due to a higher sensitivity of molecular compared to cultural assays, or to the measurement of total microbes (molecular methods) versus exclusively viable microbes (cultural methods). In fact, whereas direct CFU count detects only viable microbes, total DNA extraction and subsequent microbial genome counting does not allow investigators to distinguish between living and dead bacteria, possibly resulting in an overestimation of infectious contaminating microbes.

**Figure 6:**
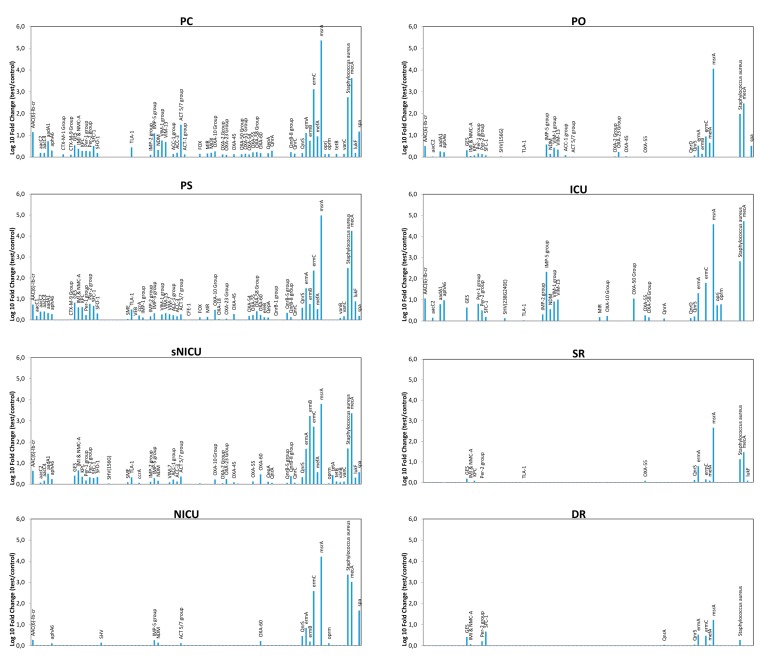
Characterization of the resistome of the contaminating microbiome in the analyzed wards, detected by qPCR microarray. Results are expressed as the mean value of Log_10_ fold change compared to negative controls, for each indicated resistance gene. PC, Pediatric Clinic; PS, Pediatric Surgery; NICU, Neonatal Intensive; sNICU, sub-Intensive Care Unit; ICU, children’s Intensive Care Unit; SR, Surgical Rooms; DR, Delivery Room; PO, Pediatric Oncology.

qPCR microarray analyses were also used to characterize the resistome of the contaminating microbial population, allowing simultaneous detection and quantification of 84 different resistance (R) genes. The results, summarized in Figure 6, revealed the presence of several genes coding antibiotic resistance in the surface microbiome. In particular, methicillin-resistance gene (*mecA*), likely ascribable to the presence of *Staphylococcus* spp., was detectable in most analyzed environments, except for DR, together with other R genes conferring resistance against macrolides (*ermA*, *ermB*), and β-lactams, including carbapenems (*VIM*-group, *OXA* group). *msrA* and *ermC* (respectively providing the resistance against macrolides and erythromycin) were also highly represented, whereas *IMP-5* group and NDM gene (New Delhi metallo-β-lactamase) were particularly represented only in the ICU ward. Since these genes are associated with the resistance to carbapenems in Gram-negative bacteria, their prevalence likely reflected the presence/selection of strains resistant to that class of antibiotics in the ICU ward. Overall, resistome analysis results paralleled the contamination level, with wards harboring high levels of R genes (PC, PS) and other, such SR and DR, where drug-resistance was barely or at all detectable in the few contaminating strains.

Comparison of the resistome detected in the contaminating population of the different wards did not reveal any statistically significant difference among PC, PS, ICU and sNICU, although a generally higher number of R genes at higher levels were observed in PC and PS wards compared to ICU and sNICU. By contrast PO, ICU and NICU grouped together as no statistically significant differences were detected among them, but they showed significantly lower resistance (*p* < 0.05) compared to PC, PS, ICU and sNICU. Lastly, SR and DR did not differ significantly from each other, but harbored a lower number and level of R genes both compared to PO-ICU-NICU group (*p* < 0.001) and to the PC-PS-ICU-sNICU group (*p* < 0.0001).

### 3.3. Environmental Analyses by NGS

**Figure 7:**
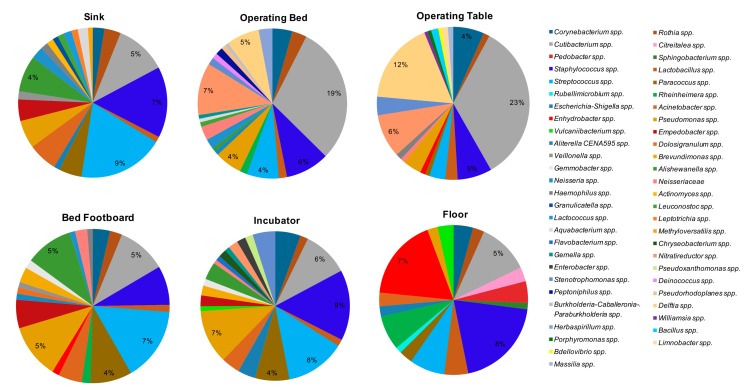
The predominant bacterial communities on tested surfaces, as detected by next-generation sequencing (NGS) analysis. Data are expressed as mean relative abundance values.

The microbial communities of the same environmental locations were analyzed in parallel by NGS. Overall, the most frequently detected bacterial genera were *Cutibacterium* spp. (formerly *Propionibacterium*), detected in 102/108 of the total analyzed samples, *Staphylococcus* spp. (100/108 samples), *Streptococcus* spp. (89/108 samples), *Corynebacterium* spp. (81/108 samples), *Pseudomonas* spp. (81/108 samples), *Paracoccus* spp. (76/108 samples), *Acinetobacter* spp. (71/108 samples), and *Rothia* spp. (64/108 samples). By grouping the samples according to the type of surface (Figure 7), the bacteria showing the highest percentages of relative abundance, compared to the other detected genera, were *Cutibacterium* spp., representing the major contaminant of operating tables (mean relative abundance 23%) and operating beds (mean relative abundance 19%), where it was present on all tested surfaces (8/8 operating beds and 8/8 operating tables). The other bacteria detected at high relative abundance on the operating tables included *Delftia* spp. (mean relative abundance 12%, in 6/8 operating tables), *Staphylococcus* spp. (mean relative abundance 5%, in 7/8 samples), *Streptococcus* spp., and *Nitrareductor* spp. (4% in 2/8 tables and 6% in 5/8 tables, respectively). In operating beds, with abundance percentages ranging from 1% to 7%, *Pseudomonas* spp. (7/8 samples), *Delftia* spp. and *Nitrareductor* spp. (6/8 samples), *Corynebacterium* spp. and *Staphylococcus* spp. (5/8 samples), and *Streptococcus* spp. (4/8 samples) were frequently observed.

*Staphylococcus* spp. represented the main contaminant of the floors (mean relative abundance 8%), where it was present in all the analyzed samples (36/36). In addition, on floors *Corynebacterium* spp. (32/36), *Cutibacterium* spp. (35/36) and *Streptococcus* spp. (32/36) were also frequently identified, with relative abundance percentages ranging from 1% to 5%. In sinks, *Streptococcus* spp. was present with the higher value of relative abundance (9%, in 27/28 sinks), together with *Staphylococcus* spp. (7%, in 27/28 samples). *Cutibacterium* spp. (24/28 samples) and *Pseudomonas* spp. (22/28 samples) were also frequently detected in this type of surface. In the incubators the main contaminants were *Pseudomonas* spp., *Staphylococcus* spp., *Cutibacterium* spp., and *Streptococcus* spp.; these last two microorganisms were found in all the incubators analyzed (8/8 samples). The means of relative abundance values were between 4% and 8%. *Streptococcus* spp. was also the bacterium showing the highest values among the bed footboards (mean relative abundance 7%).

**Figure 8:**
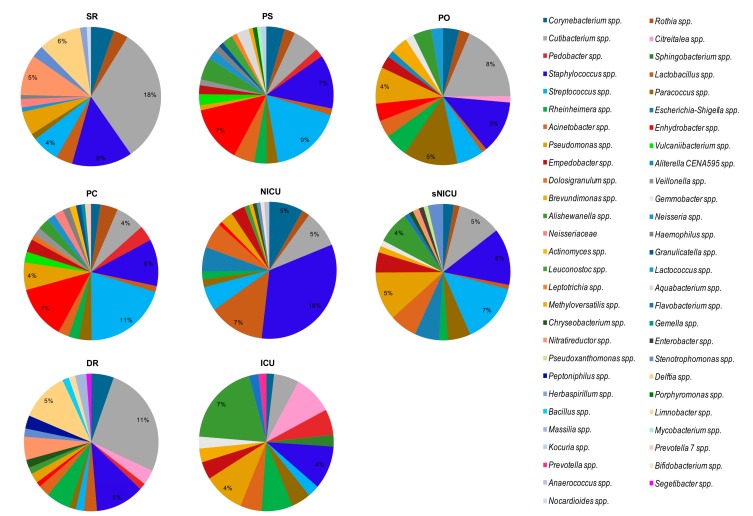
The predominant bacterial communities in analyzed wards, as detected by NGS analysis. Data are expressed as mean relative abundance values. PC, Pediatric Clinic; PS, Pediatric Surgery; NICU, Neonatal Intensive; sNICU, sub-Intensive Care Unit; ICU, children’s Intensive Care Unit; SR, Surgical Rooms; DR, Delivery Room; PO, Pediatric Oncology.

By grouping the samples on the basis of the analyzed ward (Figure 8), regardless of the type of surface analyzed, we noticed that the bacterial genera found with the highest relative abundance percentages were *Cutibacterium* spp. (18% in SR with 18/18 samples, and 11% in DR with 6/6 samples) and *Staphylococcus* spp. (18% in NICU, in 6/6 samples). In NICU, with percentages of relative abundance from 1% to 5%, *Rothia* spp., *Cutibacterium* spp., *Streptococcus* spp., and *Escherichia*–*Shigella* spp. were also identified in all the surfaces considered (6/6 samples).

Of note, in SR, *Staphylococcus* spp. and *Pseudomonas* spp. were both observed in 16/18 samples, with mean relative abundances respectively of 8% and 3%, and *Delftia* spp. was detected in 13/18 samples at 6% abundance. Analyzing the bacterial composition of the samples from PS and PC and sNICU, *Staphylococcus* spp. and *Streptococcus* spp. were the bacteria with the highest values of relative abundance (6%, 11% and 18% respectively), and in particular in sNICU were detected on 16/18 and 17/18 of surfaces analyzed. Among other bacteria frequently found in sNICU, *Acinetobacter* spp., *Pseudomonas* spp., *Cutibacterium* spp. and *Corynebacterium* spp. were detected. In ICU, the main bacterial contaminants were represented by *Acinetobacter* spp., *Cutibacterium* spp. and *Pseudomonas* spp., which were detectable in all the tested ward surfaces, with relative abundance values between 2% and 4%. In PO, again, the main contaminant was *Cutibacterium* spp. (mean relative abundance 8%), followed by *Staphylococcus* spp. and *Paracoccus* spp. (both 5% of relative abundance). As shown in Figure 7 and Figure 8, many other bacterial genera were identified, with relative abundance percentages ranging from 1% to 3%, some of which could be potential pathogens.

**Figure 9:**
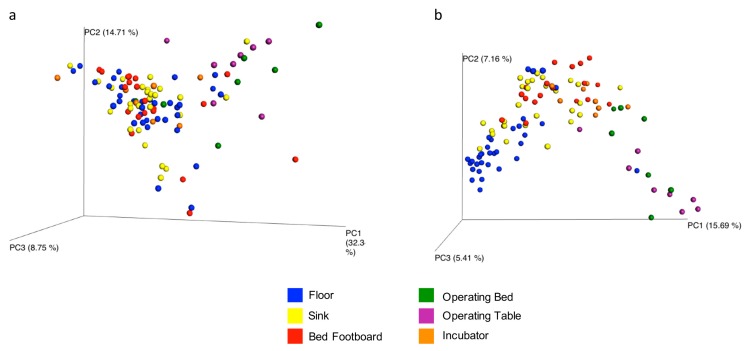
Results of UniFrac-based Principal Coordinates Analysis (PCoA) on tested surfaces. Emperor PCoA plot generated from the jackknifed_beta_diversity.py script of QIIME showing the clustering of bacterial communities according to the type of surface analyzed. Weighted (**a**) and unweighted, (**b**) UniFrac-based PCoA; each dot represents a sample.

**Figure 10:**
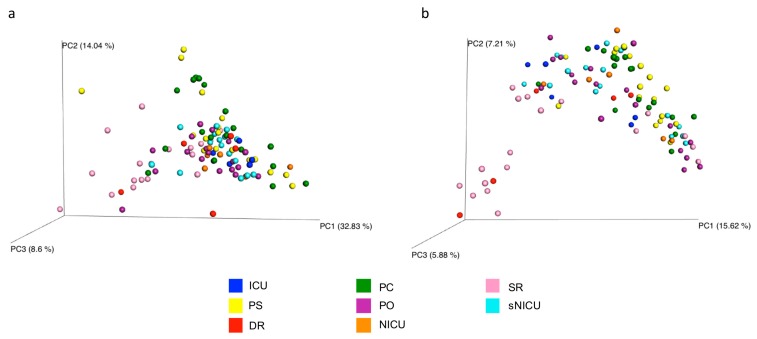
Results of UniFrac-based Principal Coordinates Analysis (PCoA) in analyzed wards. Emperor PCoA plot generated from the jackknifed_beta_diversity.py script of QIIME showing the clustering of bacterial communities according to the wards considered. Weighted (**a**) and unweighted (**b**) UniFrac-based PCoA; each dot represents a sample. PC, Pediatric Clinic; PS, Pediatric Surgery; NICU, Neonatal Intensive; sNICU, sub-Intensive Care Unit; ICU, children’s Intensive Care Unit; SR, Surgical Rooms; DR, Delivery Room; PO, Pediatric Oncology.

The overall microbial diversity comparison between samples (β-diversity) was performed using the unweighted and weighted UniFrac distance matrices. The results were visualized by a Principal Coordinates Analysis (PCoA), both with weighted and unweighted UniFrac. According to the grouping, a one-way Analysis of Similarity (ANOSIM) statistical test was applied to the UniFrac distance matrices to test for significant differences. Regarding the comparison between samples grouped by type of surface considered, ANOSIM attributed a significant difference to the grouping both for the weighted (Figure 9a) (*p* = 0.01, R = 0.21), and the unweighted UniFrac (Figure 9b) (*p* = 0.01, R = −0.47). Also as regards the grouping of the samples by ward, ANOSIM attributed a significant difference both for the weighted (Figure 10a) (*p* = 0.01, R = 0.13) and unweighted (Figure 10b) (*p* = 0.01, R = 0.14) UniFrac. These tests show there are significant statistical differences between the surfaces, in particular clustering is noted with in the floors and the operating tables.

## 4. Discussion

Recent studies show how built environments can be considered as super-organisms, with their own microbiome, highly affected by the presence of human beings [34]. For hospital environments, the kind of persisting microbiome is particularly important as it is directly correlated with the risk of acquiring healthcare-associated infections (HAIs) [35], which represent a global threat. Moreover, the increased mortality rate associated with infections sustained by drug-resistant bacteria is one of the main concerns for humankind, and it therefore is important to characterize this aspect of the contaminating microbial population, particularly in the hospital environment [36,37]. Despite the availability of highly sensitive molecular methods, including metagenomics next-generation sequencing (NGS), the study of the hospital microbiome has so far been mainly addressed by conventional culture-based methods. However, such methodologies do not have high sensitivity, are time-consuming (depending on the growth of microbial isolates on culture medium) and, mostly, their detection capacity is limited to those microbes able to grow on selected culture media, thereby missing all the unsearched taxa.

Currently, the knowledge of microbial communities colonizing the hospital environment is quite fragmented, as few studies have performed a comprehensive analysis of the hospital microbiota by comparing different methodologies. Some studies have been performed by conventional culture-based methods and others by molecular assays (qualitative PCR, real-time quantitative PCR, and later NGS), but there are no data on the direct comparison of different types of analysis performed in the same environment. In addition, despite some studies beginning to investigate different types of built-environments, including healthcare structures for adult patients [38], and adult or neonatal ICUs [39,40], there is no information about the level and type of microbial contamination in pediatric hospitals.

Based on the potential relevance of the hospital microbiome for HAI transmission, which can significantly affect patient recovery and outcomes, our study aimed to characterize the hospital microbiome by comparing conventional and molecular analyses for their potential to detail precisely the microbial communities contaminating different hospital wards and surfaces in a children’s healthcare facility, focusing on the microorganisms of primary relevance for HAIs and on their AMR.

Eight different ward/room types were analyzed, including Pediatric Clinic (PC), Pediatric Surgery (PS), Pediatric Oncology (PO), Neonatal Intensive and sub-Intensive Care Unit (NICU and NICUs), children’s Intensive Care Unit (ICU), Surgical Rooms (SR) and Delivery Room (DR). Based on the environment characteristics, sampled points included floor, bed footboard, operating bed, operating table, incubator, and sink. Each location was simultaneously sampled following two different methodologies according to subsequent microbiological (CFU count) or molecular analyses, the last including qPCR microarray and NGS analyses of microbial genera/species, and qPCR microarray analysis of the contaminating population resistome.

Overall, our results indicated important differences related to the different kinds of analysis undertaken. In general, the results concerning the bacterial species searched for using conventional or molecular qPCR microbiology showed a good level of concordance with NGS results, showing a generally higher sensitivity of molecular compared to microbiological methods.

In particular, both CFU count and qPCR evidenced a different level of contamination in different wards and surfaces, with PC being the most contaminated ward and floor/sink the most contaminated surfaces. Staphylococcal contamination was the most prevalent in all analyzed areas, and the amount of staphylococci was proportional to total contamination. However, other species of bacteria and mycetes were also easily detectable, including *Enterococcus* spp., *Enterobacteriaceae* (*K. pneumonia/Enterobacter*, *E. coli*, *A. baumannii*, *P. mirabilis)*, *P. aeruginosa*, *C, perfringens/difficile*, *C. albicans* and *A. fumigatus*. Consistently, with the sensitivity limits of the methods used, except for *Staphylococcus* spp. which were detected at high efficiency with any of the methods, the other bacterial species appeared less detectable when testing surfaces with culture-based conventional methods compared to qPCR and NGS, the latter being the most sensitive methodology. NGS analysis, in fact, beside confirmation of the high frequency of detection of *Staphylococcus* spp. (92.6% by NGS, 99.0% by qPCR and 81.5% by CFU count), further permitted identification of *Pseudomonas* spp. and *Acinetobacter* spp. at a very high efficiency (in 75% and 65.7% of samples, respectively), compared to that obtained by culture-based (7.5%) or qPCR assays (24.1%). This difference is particularly important when considering the potential of such pathogens to be associated with difficult-to-treat HAIs, especially in ICU. Beside, NGS also evidenced the presence, in almost all analyzed samples, of bacterial genera belonging to the skin/mucosa colonizer group, such as *Cutibacterium* spp. (94.4%), *Streptococcus* spp. (detected in 82.4% of samples) and *Corynebacterium* spp. (75.0% of samples). In addition, differently from the other techniques, NGS also evidenced the environmental, non-anthropic component of the hospital bacterial contamination, as it detected in most of samples environmental bacteria with low pathogenicity, including *Paracoccus* spp. (70.4% of samples), and *Rothia* spp. (59.3% samples). Based on NGS results, *Staphylococcus* and *Cutibacterium* genera represented the main contaminant of the floors, operating beds and tables. On floors also *Corynebacterium* spp. and *Streptococcus* spp. were frequently identified, whereas in sinks *Streptococcus* spp. and *Staphylococcus* spp. were the most prevalent genera, and *Cutibacterium* spp. and *Pseudomonas* spp. (22/28) were also frequently detected. The incubators appeared mostly contaminated by *Cutibacterium* spp. and *Streptococcus* spp. (both found in 100% of samples), but *Pseudomonas* spp. and *Staphylococcus* spp. were also present (with mean relative abundance values ranging between 4% to 8%). By contrast, in bed footboards *Streptococcus* spp. was the bacterium showing the highest mean values of relative abundance (7%).

Being based on the characterization of the whole bacterial population, expressed as relative abundance values, NGS generated results less superimposable with those obtained by CFU count, whereas, in general, qPCR results showed a good level of concordance with CFU count, being based on specific species detection and quantitation. The higher species-specificity of qPCR vs. NGS, however, might have potentially led to an underestimation of the presence of some pathogens, as observed for *Acinetobacter*, which was detected in 71/108 samples by NGS and 26/108 by qPCR. An overall advantage of qPCR vs. NGS consisted in providing quantitative data, depicting more closely the level of contamination in the different areas by searched pathogens. This is important when considering for example the contamination by *Staphylococcus* spp. that, although detected by NGS in almost all samples with high relative abundance values (including almost ‘sterile’ rooms such as SR and DR), had a very different load in different areas and tested surfaces (SR/DR <<< PC, PO), providing a more realistic description of the true level of contamination and of the related infectious risk.

Of note, the molecular assays were much more sensitive compared to conventional CFU count. However, the higher amount of microbes detected by molecular versus microbiological methods, might also be due to a different principle inherent in the two kinds of methodologies: CFU count only shows living cells, capable of growing in selective media, whereas molecular methods show all the strains present in the sample, being based on the extraction of total DNA from the bulk population. Thus, to avoid overestimation of infectious contamination, in future works it would be interesting to introduce differential extraction methods able to distinguish between alive and dead cells, for examples by using propidium monoazide [41], since such approach would allow a deeper understanding of the types of contamination actually able to transmit infections.

In general, NGS appears to be the only technique able to identify non-searched bacteria, and this feature renders NGS a unique tool for monitoring contaminating bacterial species, being able to identify even poorly represented species. Considering that current guidelines for hospital monitoring include only a few species among those detected by NGS, this point might be kept in mind when evaluating the safety of a hospital area. Interestingly, our NGS data were similar to those previously reported by Lax et al. [38], but dissimilar from recent data obtained in Brazilian hospitals, showing a a massive dominance of *Acinetobacter* and *Pseudomonas* in the hospital environment [42]. Overall, these differences show the potential of NGS to define very precisely the different hospital microbiomes, as proposed for confined habitats in general [43]. These data also strengthen the notion that NGS might be a useful tool when it is important to understand the impact of confinement on microbial communities, to address effectively the management of microbial contamination and associated infectious risk.

However, even 16S rRNA gene sequencing shows limitations, which should be considered when implementing it as a monitoring strategy. First, the analysis is performed on a short specific sequence, and it limits the taxonomic resolution of closely related species [44]. In addition, it is not able to distinguish between virulent or non-virulent strains, thus for accurate identification of certain pathogenic species, multiplex PCR assays or Whole Genome Sequencing (WGS) should be added. Also, 16S-based NGS cannot detect eukaryotic cells, such as mycetes, which can be very important HAI-associated pathogens. Lastly, it does not provide quantitative data, but mean relative abundances.

One further aspect that should be considered when monitoring hospital contamination is the drug-resistance of contaminating microorganisms, especially in light of the growing AMR of HAI-associated pathogens. Toward this aim, the utilized qPCR microarray analysis was able to characterize the resistome of the contaminating population, and provided results consistent with those obtained by quantitative molecular assays. In fact, the most prevalent R genes were those that can be detected in the species most prevalently found on surfaces, including the methicillin-resistance gene (*mecA*), likely linked to the presence of *Staphylococcus* spp., followed by genes conferring resistance against macrolides (*ermA*, *ermB, ermC, msrA*), β-lactams/carbapenems (*VIM*-group, *OXA* group), *IMP-5* group and *NDM* gene, differentially distributed in the different wards. Such results can thus provide a useful tool to monitor the spread of AMR in the hospital environment, and to assist design of efficient interventions and strategical countermeasures, confirming previous observations [45].

Importantly, resistome results paralleled contamination analysis results, defining diverse areas in the hospital environment, each characterized by different levels and types of contamination. Of note, some intensive care units showed contamination and resistance levels not dissimilar from PC and PS wards, highlighting the importance of contamination monitoring to prevent adverse events.

To our knowledge, our study is the first to compare directly conventional microbiology and molecular analyses for the study of hospital microbiome. Overall, based on collected data, the results of our investigation suggest that the high-throughput sequencing of 16S rRNA gene might be an effective first-step tool for monitoring the whole composition of hospital bacterial microbiome, eventually implementing amplicon sequence variants (ASV) analysis, which might improve the results [46]. Such data, together with those derived from the analysis of mycome (by 18S analysis) and of resistome/virulome, might provide a deeper knowledge of the hospital microbiome, possibly leading to significant improvements in the development of new protocols to fight HAIs and AMR.
